# Positive associations between cannabis and alcohol use polygenic risk scores and phenotypic opioid misuse among African-Americans

**DOI:** 10.1371/journal.pone.0266384

**Published:** 2022-04-08

**Authors:** Jill A. Rabinowitz, Jin Jin, Sally I-Chun Kuo, Adrian I. Campos, Miguel E. Rentería, Andrew S. Huhn, Johannes Thrul, Beth A. Reboussin, Kelly Benke, Benjamin Domingue, Nicholas S. Ialongo, Brion S. Maher, Darlene Kertes, Vanessa Troiani, George Uhl

**Affiliations:** 1 Department of Mental Health, Johns Hopkins Bloomberg School of Public Health, Baltimore, MD, United States of America; 2 Department of Biostatistics, Johns Hopkins Bloomberg School of Public Health, Baltimore, MD, United States of America; 3 Department of Psychology, Virginia Commonwealth University, Richmond, VA, United States of America; 4 Department of Genetics and Computational Biology, QIMR Berghofer Medical Research Institute, Brisbane, QLD, Australia; 5 Department of Psychiatry and Behavioral Sciences, Johns Hopkins University School of Medicine, Baltimore, MD, United States of America; 6 Sidney Kimmel Comprehensive Cancer Center at Johns Hopkins, Baltimore, MD, United States of America; 7 Centre for Alcohol Policy Research, La Trobe University, Melbourne, Australia; 8 Department of Biostatistics and Data Science, Wake Forest School of Medicine, Winston-Salem, NC, United States of America; 9 Graduate School of Education, Stanford University, Stanford, CA, United States of America; 10 Department of Psychology, University of Florida, Gainesville, FL, United States of America; 11 Geisinger, Lewisburg, PA, United States of America; 12 New Mexico VA HealthCare System, Albuquerque, New Mexico, United States of America; Boston University, UNITED STATES

## Abstract

**Background:**

This study examined whether polygenic risk scores (PRS) for lifetime cannabis and alcohol use were associated with misusing opioids, and whether sex differences existed in these relations in an urban, African-American sample.

**Methods:**

Data were drawn from three cohorts of participants (*N* = 1,103; 45% male) who were recruited in first grade as part of a series of elementary school-based, universal preventive intervention trials conducted in a Mid-Atlantic region of the U.S. In young adulthood, participants provided a DNA sample and reported on whether they had used heroin or misused prescription opioids in their lifetime. Three substance use PRS were computed based on prior GWAS: lifetime cannabis use from Pasman et al. (2018), heavy drinking indexed via maximum number of drinks from Gelernter et al. (2019), and alcohol consumption from Kranzler et al. (2019).

**Results:**

Higher PRS for lifetime cannabis use, greater heavy drinking, and greater alcohol consumption were associated with heightened risk for misusing opioids among the whole sample. Significant sex by PRS interactions were also observed such that higher PRS for heavy drinking and alcohol consumption were associated with a greater likelihood of opioid misuse among males, but not females.

**Conclusion:**

Our findings further elucidate the genetic contributions to misusing opioids by showing that the genetics of cannabis and alcohol consumption are associated with lifetime opioid misuse among young adults, though replication of our findings is needed.

## Introduction

Opioid misuse is associated with serious negative consequences, including compromised physical and mental health, emergency room visits, and overdose deaths [1–3]. In the United States, opioid misuse has reached epidemic proportions with the federal government declaring it a national emergency [4]. Within recent years, there has been a shift in the opioid epidemic with opioid overdose death rates in urban, predominantly African-American communities now exceeding rates in largely European-American, rural communities [5]. Given the tremendous public health burden associated with opioid misuse, understanding factors that confer liability for misusing opioids among urban, African-Americans may provide a greater understanding of etiological factors contributing to opioid misuse.

We previously found that alcohol and marijuana use trajectories during adolescence predicted opioid misuse in young adulthood in a largely African-American sample [6, 7]. Here, we examined whether these use patterns were partially genetically driven by examining whether lifetime cannabis and alcohol use polygenic risk score (PRS) (defined as weighted summed scores of numerous single nucleotide polymorphisms that reflect an individual’s aggregate genetic risk for a phenotype) [8, 9] are associated with phenotypic opioid misuse in an urban, African-American sample. Below, we describe research documenting a relationship between alcohol, cannabis and opioid misuse phenotypes; candidate gene and genome-wide association studies on opioid misuse; and sex differences in the genetics of opioid misuse.

### Links between phenotypic alcohol, cannabis, and opioid misuse

Individuals who misuse opioids often misuse other substances [10, 11]. Consistent with the common liability hypothesis related to substance use disorders as described by Vanyukov et al. [12], it is thought that there is a continuous latent trait, which is shared across drug classes, that is reflected in risk for developing a drug addiction. This latent trait of liability is thought to reflect an accumulation of factors including variability in genetic predispositions that drive behavior (e.g., risk taking), environmental characteristics (e.g., peer influences), culture, and the extent to which drugs are accessible [12–14]. Numerous studies have supported the common liability hypothesis. For example, using data drawn from the 2015–2017 NSDUH, Carmona et al. [11] found substantial percentages of adolescents reporting use of non-medical prescription opioids with alcohol (63%) and cannabis (52%), respectively. In addition, using Wave 1 (2001–2002) and Wave 2 (2004–2005) data drawn from the National Epidemiologic Survey of Alcohol and Related Conditions (NESARC), early adolescent alcohol use predicted opioid misuse initiation, and more frequent cannabis use predicted heavier and more frequent opioid misuse [15]. Based on previous work demonstrating a phenotypic relationship between alcohol, cannabis, and opioid use, it would seem reasonable to expect that the genetics of alcohol and cannabis are associated with misusing opioids, although whether this is the case remains unknown.

### Genetics of opioid misuse

Several candidate genes have been implicated in opioid abuse, such as *OPRM1* [16]; however, large scale genome-wide association studies (GWAS) have shown that substance use behaviors are highly polygenic [17], involving small effect sizes from single variants located throughout the genome that are not necessarily in hypothesized gene or gene regulatory regions. To date, the largest GWAS on opioid use disorder (OUD) included over 8,500 cases and 71,200 opioid-exposed controls of European ancestry. The authors found significant positive genetic correlations between OUD and alcohol consumption (*r*g = 0.38) and lifetime cannabis use (*r*g = 0.19) [17]. Similarly, other work has shown that genetic risk for illicit substance use engagement is non-specific [18]. Twin studies conducted in predominantly European ancestry samples have indicated shared genetic overlap across substance use behaviors such that prescription opioid misuse was moderately associated with cannabis use (*r*g = 0.41), with weaker associations observed with cannabis and alcohol use disorders (*r*g range = 0.07–0.15) [19]. In addition, findings from a meta-analysis revealed that an *OPRM1* variant rs1799971 (A118G) was associated with non-specific liability to substance abuse, including alcohol, cannabis, and OUD development among European ancestry individuals [20]. Given moderate genetic correlations observed across substance use phenotypes, it is possible that polygenic influences associated with one substance (e.g., cannabis) may be associated with misuse of another substance (e.g., opioids), consistent with cross-disorder work [18]. However, there is a paucity of work in this area particularly among African-Americans.

### Sex differences and the genetics of opioid misuse

Sex is an important, yet understudied factor that may influence the extent to which the same genetic variants influence use of more than one substance (e.g., polygenic risk for alcohol use predicting opioid misuse). Heritability estimates for substance use disorders have been shown to be approximately 55% for men and 73% for women [21]. There is also evidence that sex affects susceptibility to OUD with a recent gene-by-sex interaction scan indicating that the ADGRV1 rs2366929 C allele was associated with an increased risk for opioid dependence among African-American men, but not women [22]. Thus, there is reason to suspect that there may be differential associations of polygenic liability for using cannabis and alcohol with phenotypic opioid misuse, but whether this is the case remains an empirical question.

### Current study

The aims of this study were to (1) determine whether the phenotypic alcohol and cannabis use associations with opioid misuse previously observed in this sample are mirrored in their genetic associations, and (2) examine potential sex differences that may influence cannabis/alcohol use PRS–opioid misuse relations. To this end, we harnessed discoveries from some of the largest GWAS to date on cannabis and alcohol use, including lifetime cannabis use [9], heavy drinking indexed via maximum drinking [23], and alcohol consumption assessed via the AUDIT-C [24]. We specifically examined whether polygenic liabilities for these substance use phenotypes were associated with ever using heroin or misusing prescription opioids in three urban cohorts of African-American young adults. Given previous reports of genetic overlap among substance use conditions [19], we hypothesized that greater PRS for cannabis and alcohol use would be associated with misusing opioids.

## Materials and methods

### Participants

The study’s analytic sample was drawn from three cohorts of participants who were originally recruited in first grade in 1985, 1986, and 1993 as part of a series of randomized controlled trials of elementary school-based, universal prevention interventions. The trials were carried out within a single urban school district in a Mid-Atlantic region of the United States when the participants were in first grade. In terms of inclusion criteria, participants had to attend one of the participating schools, be in first grade, and be in a mainstream as opposed to a self-contained special education classroom. The interventions employed in the first two cohorts (first trial) [25] and third cohort (second trial) have been described previously [26]. Although the targets of the interventions (i.e., to reduce aggression, promote academic achievement) were the same in the first and second trials, the nature of the interventions varied. Across all three cohorts, the interventions were administered universally, or classroom-wide, and participants were followed from first grade to young adulthood. The trials and follow-up studies were approved by a University Institutional Review Board and received ethnical oversight. Prior to the age of 18, youth provided assent and their caregivers provided consent. For the current study, individuals that were youth at the onset of the study were adults and, thus, they provided informed consent. Data were anonymized before analyses were conducted.

In total, 3,110 individuals were available for recruitment in first grade, of whom 1,416 (1,103 African-Americans; 313 European-Americans) provided a successfully assayed DNA sample and completed assessments of substance use in young adulthood. Given the current study question, only African-Americans were included in our study sample (i.e., 1,103 participants).

Demographic information for the analytic sample is outlined in Table 1. Significant differences in participant sex (χ^2^ (1) = 29.36, *p* < .005), race (χ^2^ (1) = 54.72, *p* < .005, and free/reduced-priced lunch status (χ^2^ (1) = 17.48, *p* < .005) were observed between the analytic sample (i.e., 1,103 participants) and those not included in the analytic sample. In particular, there was a greater proportion of females and participants who received free/reduced-priced lunch in the analytic sample compared to those participants who were missing genetic and/or phenotypic data in young adulthood.

**Table 1:** Sample characteristics (*N* = 1,103).

Characteristic	*n* (%)
**Sex**	
Male	494 (44.8%)
Female	609 (54.2%)
**Free/Reduced-Priced Lunch** ^**a**^	
Yes	838 (79.4%)
No	217 (20.6%)
**Intervention**	
Yes	536 (48.6%)
No	567 (51.4%)
**Cohort Identification**	
Cohort 1	365 (33.1%)
Cohort 2	318 (28.8%)
Cohort 3	420 (38.1%)
**Opioid Misuse**	
Yes	84 (7.6%)
No	1,019 (92.4%)

^a^Free/reduced-priced lunch was assessed in first grade.

### Measures

#### Phenotypic opioid misuse

In young adulthood, participants reported on whether they had ever used heroin or misused prescription narcotic drugs, such as morphine, oxycodone, hydrocodone, or hydromorphone by age 30. We created variables that reflected whether participants had ever used heroin or misused prescription opioids (coded as 1) or whether they had never used heroin or misused prescription opioids in their lifetime (coded as 0).

#### Discovery GWAS samples for generating PRS

*Lifetime cannabis use*. Discovery GWAS results for lifetime cannabis use were obtained from a meta-analysis GWAS (*N* = 184,765), which included the following samples: the International Cannabis Consortium, UK Biobank, and 23and Me [9]. Across these cohorts, participants reported on whether they had ever used cannabis or marijuana (e.g., weed, dope, draw) in their lifetime. All individuals included in the GWAS were of European ancestry. Approximately 79,000 individuals had reported using cannabis or marijuana in their lifetime. Summary statistics for the 23andMe cohort were obtained under an agreement with 23andMe that protects the privacy of the 23andMe participants (http://research.23andme.com/collaborate/#dataset-access for more information).

*Alcohol use*. We created alcohol use PRS based on GWAS conducted on alcohol consumption [23] and heavy drinking [24]. Kranzler et al. [24] assessed alcohol consumption (measured via the AUDIT-C) in the Million Veterans Program (MVP) sample. The AUDIT-C is comprised of 3 items, including “How often do you have a drink containing alcohol?”; “How many standard drinks containing alcohol do you have on a typical day?”; and “How often do you have six or more drinks on one occasion?” Separate GWAS were conducted based on ancestral group. Summary statistics were obtained from dbGAP.

Gelernter et al. [23] assessed heavy drinking in the MVP sample using a single item, “In a typical month, what is/was the largest number of drinks of alcohol (beer, wine, and/or liquor) you may have had in one day?” Separate GWAS were conducted based on ancestral group (African-American GWAS N = 17,029). Summary statistics were obtained from dbGAP. We created PRS based on the African-American GWAS discovery results (N = 56,495) given that our analytic sample included only African-Americans, and research showing that performance of PRS is improved when the ancestry of the discovery sample matches the target data [27].

#### DNA extraction and preparation

In young adulthood, DNA was extracted from blood or saliva samples and was genotyped using Affymetrix 6.0 microarrays comprising 1 million single nucleotide polymorphisms (SNPs) across the genome [28]. Standard quality control steps were conducted in PLINK 2.0 [29] and implemented to ensure accurate genotypes were used in subsequent analyses. Participants with >10% missing genotype data were removed. SNPs were also removed from further analysis when they had a minor allele frequency < 0.01, missingness > 0.05, or departures from Hardy–Weinberg equilibrium at *p* < .0001. Genotypes were imputed to TopMed using the Michigan Imputation Server [30]. Resulting variants imputed with an INFO (quality) score < 0.8 were removed.

#### PRS generation

PRS were generated using the clumping and thresholding approach. Specifically, we constructed a series of scores by first applying linkage disequilibrium (LD) clumping. LD clumping is informed by LD pruning as it identifies the most significant genetic associations in an LD window and filters out SNPs with low genetic correlations [31]. The r^2^ threshold (i.e., the absolute value of the pairwise correlation between SNPs) was set to 0.1, 0.2, 0.4, 0.6, 0.8 or 1 to keep SNPs that have absolute pairwise correlations weaker than r^2^ within an LD window of 2 megabases [31]. This LD clumping step was conducted in PLINK 2.0 [29] based on the 1000 Genomes Phase 3 Reference Panel described in further detail below [32]. Next, we selected from the remaining set of SNPs, specifically the ones that reached the *p*-value significance thresholds of *p* = 5×10^−1^, 5×10^−2^, 5×10^−4^, 5×10^−6^, or 5×10^−8^. Scores were constructed as weighted sums of the effect alleles of the selected SNPs, with the weights equal to the effect size estimated from the discovery GWAS.

To create lifetime cannabis use PRS based on European ancestry GWAS, we first tuned r^2^ and *p* over the grid of values with respect to the prediction accuracy among the 313 individuals of European ancestry in the larger sample from which the study sample was drawn. Based on these analyses, we report performance of the selected optimal score on the 1,103 validation individuals of African-American ancestry. Regarding the alcohol use PRS, which were created based on GWAS conducted in African-American samples, we report two-fold cross-validated performance on the 1,103 validation sample of individuals of African-African ancestry. Specifically, we split the validation sample of individuals into two folds; first, we tuned r^2^ and *p* with respect to the prediction accuracy among individuals in the first fold and calculated the corresponding scores for the individuals in the second fold; second, we tuned r^2^ and *p* with respect to the prediction accuracy among individuals in the second fold and calculated the corresponding scores for the individuals in the first fold. We then report metrics for the predictive power of the calculated scores from the two-fold cross-validation.

Our LD reference panel was constructed using the Genome Reference Consortium Human Build 38 (GRCh38) for the 1000 Genomes Phase 3 that reflects unrelated individuals of the same ancestry as the discovery GWAS. When constructing the PRS for lifetime cannabis use, we selected approximately 1.1 million HapMap3 SNPs with MAF > 0.01 that were available in the 1000 Genomes Phase 3 Reference Panel. When constructing PRS for alcohol consumption and heavy drinking (which were based on GWAS of individuals of African-African ancestry), we used all SNPs available from these GWAS that were also in the 1000 Genomes Phase 3 Reference Panel; this led to approximately 2.3 million SNPs for the alcohol consumption PRS and 1.4 million SNPs for the heavy drinking PRS. Principal components analysis was used to create the population stratification control variables in PLINK 2.0 [29]. This process uses an orthogonal transformation to reduce the multi-dimensional genome-wide SNP data into a smaller number of genetic ancestry principal components (PCs). After pruning the target data (i.e., our analytic sample), PCs were generated, the first 10 of which were used to account for population stratification.

### Statistical analyses

A series of logistic regressions were conducted in R [29] to examine whether the lifetime cannabis use and alcohol use PRS (i.e., alcohol consumption, heavy drinking) were associated with ever misusing opioids. Results involving the main effects of participant sex, cohort, intervention status, and PCs on phenotypic opioid misuse can be found in S1 Table. To evaluate model fit of the logistic regressions, we performed Hosmer-Lemeshow tests [33]. Across models, we report area under the ROC curve (AUC), a metric ranging from 0.5 (complete absence of separation) to 1 (perfect separation), which indicates the probability that the model will correctly categorize individuals [34]. We also report sensitivity corresponding to 80% specificity (S80) and pseudo *R*^*2*^ for the regression analyses.

Three separate analyses were conducted for each PRS (i.e., one for lifetime cannabis use, one for alcohol consumption, and one for heavy drinking). In analyses involving the whole sample, participant sex was included as a covariate given sex differences in drug use that have been observed across the literature [35]. Cohort was also included to adjust for potential effects of recruitment year. Intervention status was also controlled for given that participation in the interventions has been associated with reduced risk for substance use among adolescents [36].

To examine potential sex differences, we conducted sex by PRS interactions. As with the primary analyses, three separate analyses were conducted involving the interaction between participant sex and each PRS. In these models, participant demographics and relevant PRS were included as covariates. We also conducted sex-stratified analyses to determine whether sex differences existed between the lifetime cannabis use and alcohol use PRS in relation to ever misusing opioids. For these analyses, intervention status, cohort, and the first 10 genetic ancestry PCs were included as covariates. Last, we conducted a power analysis in the full and sex-limited samples to determine our ability to detect significant associations at an alpha of 0.05.

## Results

As shown in Table 1, approximately 8% of African-Americans (*n* = 84) in young adulthood reported ever misusing opioids. Close to 8% of males (*n* = 40) and 7% of females (*n* = 44) reported misusing opioids in their lifetime. There was a small, positive correlation between the lifetime cannabis use PRS and the (a) heavy drinking PRS (*r* = 0.10), and (b) alcohol consumption PRS (*r* = 0.07). A strong, positive correlation was observed between the heavy drinking PRS and the alcohol consumption PRS (*r* = 0.90).

Results from our power analyses indicate that in the full sample (*N* = 1,103), we have 80% power to detect an odds ratio (OR) of 1.38 given 84 cases of opioid misuse. In addition, given the endorsement rates of lifetime opioid misuse among males (*n* = 40) and females (*n* = 44), we have 80% power to detect (a) an OR of 1.59 among males, and (b) an OR of 1.55 among females. Results from the logistic regression analyses are presented below involving the whole sample followed by the sex interaction and sex-stratified analyses.

### Whole sample

The Hosmer-Lemeshow goodness-of-fit test showed no evidence of poor fit for any of the regression models (*p*-values ranging from 0.557–0.748). The model that included only the covariates (i.e., sex, intervention status, cohort, and the 10 ancestry specific PCs) had an AUC of 0.60, an S80 equal to 0.32, and a pseudo *R*^2^ equal to 4.5%. None of the covariates were significant (*p*-value range = 0.21–0.92), except for PC9 (*p* = 0.039).

The lifetime cannabis use PRS was significantly positively associated with opioid misuse (aOR = 1.33, 95% CI: 1.05, 1.69, *p* = 0.017) such that greater polygenic risk for cannabis use was linked to greater risk for misusing opioids (Table 2). Following the addition of the lifetime cannabis use PRS, the AUC increased to 0.64, the S80 remained equal to 0.32, and *R*^*2*^ increased by 1.2%. Similarly, a higher PRS for alcohol consumption was associated with an increased risk for misusing opioids (aOR = 1.30, 95% CI: 1.04, 1.64, *p* = 0.023); the addition of this PRS resulted in the AUC to increase to 0.62, the S80 to increase to 0.41, and *R*^*2*^ to increase by 1.1%. Finally, a higher heavy drinking PRS was associated with a greater likelihood of misusing opioids (aOR = 1.31, 95% CI: 1.06, 1.62, *p* = 0.013). The inclusion of the heavy drinking PRS increased the AUC to 0.63, the S80 to 0.41, and *R*^*2*^ by 1.3%.

**Table 2:** Cannabis and alcohol consumption PRS associations with the likelihood of ever misusing opioids in the whole sample (*N* = 1,103).

PRS	Opioid Misuse				
	aOR (95% CI)	*p*	AUC	S80	△ *R*^2^ (%)
Lifetime cannabis use PRS	1.33 (1.05, 1.69)	0.017	0.64	0.32	1.2%
Heavy drinking PRS	1.31 (1.06, 1.62)	0.013	0.63	0.41	1.3%
Alcohol consumption PRS	1.30 (1.04, 1.64)	0.023	0.62	0.41	1.1%

Note. Participant sex, intervention status, cohort, and 10 ancestry-specific principal components were included as covariates.

AUC = area under the curve; S80 = sensitivity corresponding to 80% specificity.

### Sex x prs interaction analyses

Next, we conducted a series of two-way interactions involving participant sex and each PRS. Across regression analyses, the Hosmer-Lemeshow goodness-of-fit test showed no evidence of poor fit (*p*-value range = 0.087–0.718). The interaction between sex and the lifetime cannabis use PRS was not significant (aOR = 1.56, 95% CI: 0.77, 3.17, *p* = 0.216), although point estimates suggest a stronger association between genetic risk for lifetime cannabis use and opioid misuse among males compared to females. The addition of the interaction term did not result in changes in the AUC or S80, but contributed to an *R*^2^ increase of 0.05%.

The sex by alcohol consumption PRS was significant (aOR = 2.71, 95% CI: 1.27, 5.79, *p* = 0.013) such that males with greater polygenic risk for alcohol consumption were at heightened risk for opioid misuse. Relative to the model with the covariates and alcohol consumption PRS, the addition of the sex by alcohol consumption interaction term resulted in the AUC to increase to 0.64, the S80 to remain the same (equal to 0.38), and the *R*^2^ to increase by 0.07%.

Significant interactions were observed between sex and the heavy drinking PRS (aOR = 2.33, 95% CI: 1.18, 4.67, *p* = 0.014) such that males with a higher heavy drinking PRS were more likely to misuse opioids. Compared to the model with just the covariates and heavy drinking PRS, the addition of the sex by heavy drinking PRS interaction term resulted in the AUC increasing to 0.64, the S80 remaining the same (equal to 0.38), and the *R*^2^ increasing by 0.07%,

### Sex-stratified analyses

In regression analyses conducted among males and females, the Hosmer-Lemeshow goodness-of-fit test indicated no evidence of poor fit (*p*-values range = 0.190–0.926). Among males, the covariate-only model had an AUC of 0.61, an S80 equal of 0.40, and an *R*^2^ of 2.7%. None of the covariates were significant (*p*-value range = 0.14–0.93). As shown in Table 3, the lifetime cannabis use PRS was significantly positively associated with opioid misuse (aOR = 1.56, 95% CI: 1.10, 2.22, *p* = 0.014). The inclusion of the lifetime cannabis use PRS increased the AUC to 0.67, decreased S80 to 0.38, and increased *R*^2^ by 2.9%. Similarly, the heavy drinking PRS was significantly positively associated with opioid misuse (aOR = 1.68, 95% CI: 1.18, 2.39, *p* = 0.004); the addition of this PRS increased the AUC to 0.67, the S80 remained the same (equal to 0.40), and increased the *R*^2^ by 3.9%. Last, a higher PRS for alcohol consumption was associated with heightened risk for misusing opioids (aOR = 1.68, 95% CI: 1.18, 2.39, *p* = 0.004); the addition of the alcohol consumption PRS increased the AUC to 0.67, the S80 remained the same (equal to 0.40), and the *R*^2^ increased by 4.1%.

**Table 3:** Sex-stratified analyses involving associations between the cannabis and alcohol consumption PRS with the likelihood of ever misusing opioids.

PRS	Opioid Misuse				
	aOR (95% CI)	*p*	AUC	S80	△ *R*^2^ (%)
***Males***					
Lifetime cannabis use PRS	1.56 (1.10, 2.21)	0.013	0.67	0.38	2.9%
Heavy drinking PRS	1.59 (1.17, 2.17)	0.003	0.67	0.40	3.9%
Alcohol consumption PRS	1.68 (1.18, 2.39)	0.004	0.67	0.40	4.1%
***Females***					
Lifetime cannabis use PRS	1.21 (0.87, 1.67)	0.261	0.64	0.38	0.50%
Heavy drinking PRS	1.08 (0.79, 1.47)	0.627	0.63	0.38	0.09%
Alcohol consumption PRS	1.06 (0.77, 1.45)	0.718	0.63	0.38	0.05%

Note. Intervention status, cohort, and 10 ancestry-specific principal components were included as covariates.

AUC = area under the curve; S80 = sensitivity corresponding to 80% specificity.

Among females, the covariate-only model had an AUC of 0.64, the S80 was equal to 0.38, and had an *R*^2^ of 6.3%. PC2 was significantly associated with opioid misuse (*p =* 0.012); however, none of the other covariates were significant (*p*-value range *=* 0.071–0.99). PRS for lifetime cannabis use (aOR = 1.21, 95% CI: 0.87, 1.67, *p* = 0.261), alcohol consumption (aOR = 1.06, 95% CI: 0.77, 1.45, *p* = 0.718), and heavy drinking (aOR = 1.36, 95% CI: 0.82, 2.27, *p* = 0.237) were not significantly associated with opioid misuse. The addition of these PRS did not improve AUC, S80, or result in the *R*^2^ to increase (see Table 3).

## Discussion

The increasing prevalence of heroin and misuse of prescription opioids represents a growing public health concern, with alarming increases in opioid-related negative outcomes including overdose and mortality [2, 4]. Whereas several studies have observed positive phenotypic associations between cannabis, alcohol, and opioid misuse [6, 7, 11], less is known about whether genetic liability for cannabis and alcohol use is associated with phenotypic opioid misuse. In the present study, we sought to determine whether the phenotypic alcohol and cannabis use associations with opioid misuse observed in our prior studies could be explained, in part, by a common genetic liability. To that end, we used recent large-scale GWAS of cannabis and alcohol use and examined whether cannabis and alcohol use PRS were associated with ever misusing opioids in young adulthood in an urban sample of African-Americans.

We found that an approximately 1.3-fold increase in the odds of misusing opioids was associated with a one unit increase in the cannabis use PRS in the whole sample, and an approximately 1.5-fold increase in the odds of misusing opioids among males (in sex-stratified analyses only). These findings parallel previous phenotypic findings showing a positive relationship between phenotypic cannabis and opioid misuse [6, 7] and nationally representative samples [11]. The association between polygenic liability for lifetime cannabis use and phenotypic opioid misuse may be a reflection of similar neurobiological system functioning and overlapping expression of endocannabinoid and opioidergic systems [37]. For example, both endocannabinoid and opioid signaling play a role in reward and reinforcement [37] and there is evidence that increased cannabinoid administration increases endogenous opioid levels and vice versa [38]. Moreover, it is possible that greater polygenic liability for using cannabis may be associated with personality traits (i.e., behavioral disinhibition) or psychological symptoms (i.e., externalizing behaviors) [39] that confer risk for misusing opioids. Future research should explore potential pathways and mechanisms through which genetic predispositions for using cannabis exacerbate risk for misusing opioids.

An approximately 1.3-fold increase in the odds of misusing opioids was associated with a one unit increase in alcohol use (both consumption and heavy drinking) PRS in the whole sample, and an approximately 1.7-fold increase in the odds of misusing opioids among males specifically. These findings are consistent with research indicating positive phenotypic associations between alcohol consumption and opioid misuse observed previously [7], as well as with work indicating that binge drinkers are twice as likely to endorse prescription drug misuse [40]. Findings from candidate gene studies suggest that individuals with alcohol and opioid dependence are more likely to have shared genetic variants and be homozygous (A1/A1) or heterozygous (A1/A2) for the dopamine receptor coding gene (DRD2) [41]. Moreover, both alcohol and opioid use have been implicated in similar neurobiological changes, including the release of endogenous opioids (e.g., β-endorphins), which results in increased dopamine signaling in the mesolimbic reward system [42]. Shared risk factors such as comorbid psychiatric disorders, externalizing behaviors, or a family history of substance use may reflect elevated genetic liability for alcohol use and partially account for the positive association between polygenic liability for alcohol use and phenotypic opioid misuse [42, 43], though future research in this area is needed.

There are several explanations to account for sex differences in associations between the PRS and phenotypic opioid misuse. First, the positive association between phenotypic opioid misuse and both alcohol use PRS may be due to the nature of the discovery samples for these PRS. Indeed, the GWAS involving alcohol consumption and heavy drinking were conducted using the MVP sample, a predominantly male sample of veterans [44]. Thus, the effect estimates of individual genetic variants in the discovery GWAS may more closely resemble the effects in males rather than females. Recent work has shown that genetic variants associated with substance use may differ depending on sex [22], further highlighting that the GWAS discovery sample characteristics may affect the associations observed in other samples. Second, the effects of environmental factors on opioid misuse may vary as a function of gender, with some work suggesting that greater exposure to poverty and lower socioeconomic status were associated with greater prescription opioid misuse among women, but not men [45]. Moreover, women seeking treatment for opioid use disorder are more likely than men to have experienced trauma, including intimate partner violence, and also evidence greater psychiatric comorbidities including anxiety and depression [46]; thus, women may be more likely to self-medicate with opioids in response to stressors than men [47]. Finally, there are also a number of hormonal factors that might explain differential opioid misuse in women compared to men, including that opioid receptor binding, coupling, and density vary as a function of endogenous opioid and stress hormone systems, and that ovarian hormones might directly impact these interactions in women [48]. Future work on the complex relationship between genetics, contextual factors, hormone signaling, and the development of opioid misuse in women is needed.

There are some limitations of the current study. First, although we used summary statistics drawn from GWAS of alcohol consumption that were conducted in samples of individuals of African-American ancestry, discovery statistics on lifetime cannabis use for African-American ancestry individuals were not available. As noted previously, the performance of PRS is more accurate when the ancestry of the discovery sample matches the ancestry of the target sample due to differences in allele frequencies and linkage disequilibrium [27]. Thus, recruitment of more diverse ancestral samples that better capture genetic diversity, as well as the development of methodologies that improve the portability of PRS across ancestries, are needed. Second, although we used large-scale alcohol use GWAS that were conducted using African-American individuals, these discovery samples included predominantly male, older adults. Future gene identification efforts should aim to recruit more representative samples.

## Conclusions

Our study is the first to examine polygenic risk for lifetime cannabis use and alcohol use in relation to phenotypic opioid misuse in an African-American sample. While our results highlight that alcohol and cannabis PRS are linked to misusing opioids, using PRS for risk stratification or for informing physician prescribing decisions is not warranted at this time due, in part, to the low current predictive validity of such measures and to practical limitations with respect to prevention or early intervention among those deemed to be at “high genetic risk” [49]. For example, it is not clear how a patient with a substance use disorder or a young adult experimenting with recreational drugs would act upon the knowledge of their own “genetic risk for addiction.” However, such metrics may in the future allow clinicians to stratify patients in treatment (e.g., those at “low” genetic risk may achieve abstinence with low intensity therapy), and genetic variants comprising PRS may highlight specific biological pathways for future pharmacotherapeutic interventions for use among those seeking treatment.

## Supporting information

S1 Table Main effects of the covariates in predicting phenotypic opioid misuse in the whole sample and among males and females. (DOCX)
