# Supplementary material for: Positive associations between cannabis and alcohol use polygenic risk scores and phenotypic opioid misuse among African-Americans
Source: PLoS One. 2022 Apr 8;17(4):e0266384. doi: 10.1371/journal.pone.0266384 (PMC8993003; doi:10.1371/journal.pone.0266384)
Supplement: S1 Table — (DOCX) [file pone.0266384.s001.docx]

**Supplementary Table 1. Main effects of the covariates in predicting phenotypic opioid misuse in the whole sample and among males and females**

| **Whole Sample** |  |  |
| --- | --- | --- |
| **Covariate** | **OR (95% CI)** | ***p*** |
| Gender | 0.88 (0.54, 1.40) | 0.59 |
| Intervention status | 1.3 (0.80, 2.10) | 0.29 |
| Cohort | 1.2 (0.91, 1.6) | 0.18 |
| PC1 | 1.90^^2^ (1.0^^-15^, 3.5^^19^) | 0.80 |
| PC2 | 1.27^^40^ (2.3^^-3^, 6.0^^82^) | 0.66 |
| PC3 | 2.7^^3^ (2.2^^-3^, 3.5^^19^) | 0.92 |
| PC4 | 5.4^^18^ (2.6^^-68^, 1.1^^33^) | 0.50 |
| PC5 | 1.9^^5^ (1.2^^-17^, 2.9^^27^) | 0.64 |
| PC6 | 3.8^^-9^ (1.4^^-28^, 1.0^^11^) | 0.40 |
| PC7 | 3.1 (1.1^^-11^, 9.1^^11^) | 0.93 |
| PC8 | 2.5^^-2^ (1.1^^-16^, 5.6^^12^) | 0.83 |
| PC9 | 2.6^^23^ (17, 3.9^^45^) | 0.39 |
| PC10 | 1.4^^11^ (5.9^^-7^, 3.5^^28^) | 0.21 |
| **Males** |  |  |
| **Covariate** | **OR (95% CI)** | ***p*** |
| Intervention status | 1.2 (0.60, 2.5) | 0.59 |
| Cohort | 1.1 (0.70, 1.7) | 0.73 |
| PC1 | 1.5^^8^ (6.1^^-19^, 3.7^^34^) | 0.54 |
| PC2 | 1.1^^3^ (2.4^^-59^, 5.3^^65^) | 0.92 |
| PC3 | 1.5^^6^ (2.1^^-64^, 1.1^^76^) | 0.86 |
| PC4 | 4.1^^-23^ (5.4^^-95^, 3.1^^49^) | 0.54 |
| PC5 | 1.0^^-4^ (7.2^^-40^, 1.4^^31^) | 0.82 |
| PC6 | 5.5^^-2^ (1.5^^9^, 2.1^^26^) | 0.93 |
| PC7 | 4.8^^12^ (2.3^^-10^, 1.0^^35^) | 0.27 |
| PC8 | 1.4^^-2^ (4.6^^-26^, 4.2^^21^) | 0.88 |
| PC9 | 1.0^^15^ (1.9^^-20^, 5.9^^49^) | 0.40 |
| PC10 | 7.0^^23^ (1.2^^-8^, 4.1^^55^) | 0.14 |
| **Females** |  |  |
| **Covariates** | **OR (95% CI)** | ***p*** |
| Intervention status | 1.4 (0.72, 2.8) | 0.31 |
| Cohort | 1.3 (0.84, 2.8) | 0.26 |
| PC1 | 0.68 (1.1^^-25^, 4.2^^24^) | 0.99 |
| PC2 | 2.7^^79^ (1.5^^17^, 4.9^^141^) | 0.12 |
| PC3 | 1.0^^-18^ (5.8^^-87^, 1.8^^50^) | 0.61 |
| PC4 | 1.5^^-21^ (4.8^^-94^, 5.0^^51^) | 0.57 |
| PC5 | 1.3^^13^ (5.6^^-19^, 3.2^^44^) | 0.41 |
| PC6 | 3.8^^-15^ (4.1^^-43^, 3.6^^13^) | 0.31 |
| PC7 | 9.2^^-4^ (5.2^^-21^, 1.6^^14^) | 0.73 |
| PC8 | 4.8^^-6^ (8.0^^-27^, 2.9^^15^) | 0.62 |
| PC9 | 2.6^^28^ (3.9^^-3^, 1.7^^59^) | 0.71 |
| PC10 | 2.8 (4.4^^-30^, 1.8^^30^) | 0.98 |
